# Metabolic Regulation in the Maintenance of *Drosophila* Testis Stem Cells

**DOI:** 10.3390/ijms27041884

**Published:** 2026-02-15

**Authors:** Jiao Liu, Peixin Xu, Yichen Liu, Yuke Xie, Zixuan Liu, Gyeong Hun Baeg

**Affiliations:** Faculty of Health Sciences, University of Macau, Taipa, Macau 999078, China

**Keywords:** *Drosophila melanogaster*, genetics, germline stem cells, metabolic regulation, signaling pathways

## Abstract

Stem cells maintain tissue homeostasis through precisely regulated self-renewal and differentiation, processes largely dependent on metabolic control. The *Drosophila* testis provides an ideal model system to study metabolism regulation of stem cell homeostasis due to many advantages, including its well-defined stem cell niche architecture and genetic tractability. Recent studies have revealed that germline stem cells (GSCs) and somatic cyst stem cells (CySCs) exhibit distinct metabolic profiles. In particular, GSCs exhibit a metabolic feature closely associated with mitochondrial dynamics, lipid metabolism, and redox homeostasis, all of which are essential for maintaining their stem identity through the regulation of TOR (Target of Rapamycin) signaling. Nutrient sensing through the insulin/TOR, BMP, and JAK-STAT pathways integrates nutritional cues with developmental programs. Lipid metabolism and membrane homeostasis further contribute to the maintenance of stem cells. Metabolic intermediates function as signaling molecules, modulating niche-stem cell interactions and epigenetic modifications in stem cells. Hence, dysregulation of metabolic homeostasis can lead to stem cell depletion and age-related reproductive decline. This review synthesizes the current understanding of metabolic regulation in *Drosophila* testis stem cell maintenance, identifies critical knowledge gaps, and explores future research directions such as spatial/temporal metabolomics approaches. Lastly, we highlight how these insights may help understand mammalian stem cell biology and regenerative medicine.

## 1. Introduction

Stem cells represent a unique cell population capable of long-term self-renewal while maintaining potential to generate differentiated progeny [1]. Unlike differentiated cells, which are metabolically optimized for specific functions, stem cells must balance energy production for biosynthetic demands required for rapid proliferation and maintenance of stemness [2]. Over the past two decades, metabolism has emerged as a fundamental regulator of stem cell fate decision rather than merely a consequence of cellular state [3]. Metabolic pathways not only provide ATP and biosynthetic intermediates but also generate signaling molecules that directly influence gene expression, chromatin architecture, and protein modifications in stem cells. In particular, the metabolic–epigenetic axis has profound implications for understanding how stem cells respond to environmental cues and maintain tissue homeostasis throughout an organism’s lifespan [2].

**Figure 1:**
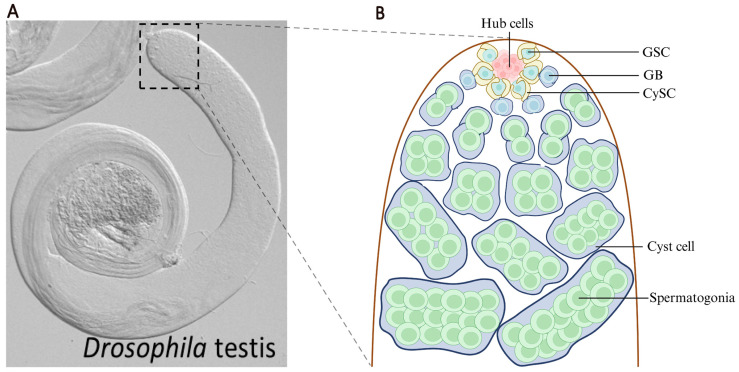
Stem cell niche in the *Drosophila* testis. (**A**) The *Drosophila* testis is a blunt-ended, coiled structure. Germline stem cells (GSCs) and somatic cyst stem cells (CySCs) reside at the apical tip of testis. (**B**) Schematic of the *Drosophila* testis. GSCs divide to produce gonialblasts (GBs), which undergo differentiation into spermatogonia. A pair of CySCs encapsulate each GSC and regulate GSC development. CySCs divide to form cyst cells. Each GB is enclosed by a pair of cyst cells, creating a functional unit for spermatogenesis.

The *Drosophila* testis provides an unparalleled experimental platform for investigating stem cell biology due to its simple and well-characterized cellular organization, with powerful genetic tools and reagents [4]. The *Drosophila* testis is a blunt-ended, coiled tube (Figure 1A). At the apical tip of each testis lies a cluster of post-mitotic somatic cells, known as hub cells (8–15 cells). Typically, 6–10 germline stem cells (GSCs) are tightly anchored to the hub, and a pair of somatic cyst stem cells (CySCs) surround a single GSC to support the development of GSC [5] (Figure 1B). A prototypical *Drosophila* testis stem cell niche/microenvironment is thus made up of hub cells and the two stem cell populations. This anatomically well-defined niche allows for precise visualization and manipulation of stem cell populations in their native microenvironment [6]. The conservation of key signaling pathways such as TGF-β/BMP (Bone Morphogenic Protein), JAK-STAT, and Notch between *Drosophila* and mammals makes findings from the *Drosophila* system highly translatable. Moreover, the testis displays robust responses to nutritional signals and aging, making it ideal for studying how metabolism integrates systemic cues to regulate homeostasis of local stem cell populations [7].

Research on *Drosophila* testis stem cells has evolved through distinct phases. Initial studies in the 1990s and early 2000s focused on identifying niche components and characterizing asymmetric divisions that maintain stem cell populations [5]. The discovery of the hub as a signaling center and elucidation of the BMP and JAK-STAT pathway function in the early 2000s have established the fundamental principles of stem cell niche biology. Subsequently, attention has shifted to understanding how extrinsic signals from the niche coordinate with intrinsic factors to control stem cell behavior [5]. In recent years, advances of technology such as single-cell transcriptomics, live imaging systems, and metabolomics have provided further insights into cellular heterogeneity and regulatory mechanisms in testis stem cells, particularly highlighting the central role of metabolism in coordinating stem cell fate decision with organismal physiology [8].

In this review, we systematically describe major metabolic features of *Drosophila* testis stem cells, including glycolysis, oxidative phosphorylation (OXPHOS), mitochondrial function, and metabolic crosstalk between stem cells and their microenvironment. We then introduce the key signaling pathways, including TGF-β/BMP, JAK-STAT, and Insulin/TOR, that function to govern these processes. Finally, we discuss potential applications of artificial intelligence (AI), particularly convolutional neural networks (CNNs) and transfer learning (TL), in dissecting metabolic networks and advancing stem cell metabolism research.

## 2. Metabolic Profiles and Characteristics of Testis Stem Cells in *Drosophila*

### 2.1. Glycolysis–OXPHOS Balance in GSCs and CySCs

GSCs and somatic CySCs in the adult *Drosophila* testis exhibit distinct yet coordinated metabolic profiles that are fundamental to their maintenance and function. GSCs in *Drosophila* testis exhibit relatively low intrinsic metabolic activity and rely on extrinsic nutrient availability, which includes systemic inputs regulated by insulin/TOR signaling [9]. GSCs rely relatively less on autonomous OXPHOS for energy production, but instead exhibit a clear metabolic compartmentalization with its somatic niche [10]. However, as cell fate shifts towards differentiation, metabolic patterns undergo significant changes. Transcriptome sequencing data revealed that transcripts of several ubiquitously expressed OXPHOS-related genes are present at higher levels in progenies differentiated from GSCs. For instance, *SDHC* (Succinate Dehydrogenase Complex Subunit C), a key gene of tricarboxylic acid (TCA) cycle, shows a high transcript abundance in early spermatogonial stage, and *Irp-1A* (Iron Regulatory Protein 1A) shows a similar enrichment in the spermatocyte stage, suggesting that enhanced mitochondrial metabolic activity is a key feature of early germ cell differentiation [11]. Similarly, in *Drosophila* ovaries, gene expression analysis indicates that young GSCs are in a metabolically quiescent state, with extremely low expression levels of genes related to the TCA cycle and glycolysis. For example, transcripts for the key TCA enzyme Oxoglutarate dehydrogenase and the glycolytic enzyme Aldolase are barely detectable in young female GSCs [12]. This suggests that young female GSCs do not primarily rely on endogenous oxidative metabolism for energy supply. Notably, in cystoblasts and early cyst stages, mitochondrial activity and metabolic activity levels significantly increase [13]. Therefore, in both *Drosophila* gonads, a transition from GSCs to differentiated germ cells is accompanied by a significant reprogramming of energy metabolic modes. This process involves a progressive increase in mitochondrial activity and oxidative metabolic capacity, and such metabolic remodeling is thought to support the elevated energy demands during differentiation.

Unlike *Drosophila* stem cells, which exhibit low metabolic activity, in some mammals, such as mice, spermatogonial stem cells display high glycolytic activity even under normoxic conditions, reminiscent of the Warburg effect observed in cancer cells and embryonic stem cells [14]. This glycolytic bias in spermatogonial stem cells appears essential for maintaining rapid ATP generation and providing biosynthetic precursors for nucleotide and amino acid synthesis required for their cell division. This metabolic preference is not merely a consequence of cellular state but actively contributes to maintenance of stem cell identity and niche residence. Mouse spermatogonial stem cells (SSCs) tend to favor high glycolytic metabolism, whereas *Drosophila* GSCs exhibit greater metabolic flexibility. This difference may arise from distinct testicular structures and microenvironmental regulatory mechanisms in the two species. From an evolutionary perspective, mammalian testes have a higher degree of vascularization. In mouse testis, the blood–testis barrier separates the seminiferous epithelium into two compartments: the basal and adluminal compartments [15]. The SSC niche is located in the avascular basal compartment of seminiferous tubules, resulting in a hypoxic state [16]. This structural organization may activate the HIF-1α signaling pathway and upregulate the expression of glycolytic enzymes, thereby providing a physiological basis for the preference of mouse SSCs for a highly glycolytic metabolic mode reliant on anaerobic energy production. Mechanistically, under normoxic conditions, HIF-1α is modified by prolyl hydroxylase domain (PHD) enzymes and rapidly degraded through the von Hippel–Lindau (VHL)-mediated ubiquitin–proteasome pathway. Under hypoxic conditions, this process is inhibited, allowing HIF-1α to accumulate and translocate to the nucleus, where it forms a heterodimer with HIF-1β [17]. The HIF-1 complex then transcriptionally activates multiple glycolysis-related genes by binding to hypoxia-responsive elements in the promoters of target genes, such as *GLUT1* and *LDHA* [18,19]. HIF-1α-mediated glycolytic metabolic reprogramming has been well documented in multiple experimental models, such as mouse osteoclasts. At present, its potential role in the SSC niche is mainly inferred from the relatively hypoxic environment of the basal compartment in the mouse testis, the previously observed glycolysis-biased metabolic features, and the conserved nature of hypoxia signaling pathways.

In contrast, *Drosophila* testis is a simple tubular structure that relies on an open circulatory system, lacking the dense capillary networks found in mammals, with nutrients primarily diffusing to tissues via hemolymph [20]. Oxygen is primarily delivered directly by the tracheal system in *Drosophila* [21]. Therefore, there is no distinct local oxygen gradient in *Drosophila* testis comparable to that in mammals. The maintenance and metabolic state of *Drosophila* male GSCs are more regulated by systemic nutrient signals, exhibiting greater metabolic flexibility. Although substantial differences exist between *Drosophila* and mammals in testicular architecture, vascularization, and stem cell niche organization, fundamental principles governing stem cell maintenance and metabolism are evolutionarily well conserved. Notably, it has been reported that approximately 96% of genes identified as essential for GSC maintenance in *Drosophila* have human homologs [22]. Therefore, while distinct microenvironmental conditions may give rise to divergent metabolic phenotypes in mammalian SSCs and *Drosophila* GSCs, the core regulatory logic underlying stem cell maintenance is likely conserved across vertebrate and invertebrate systems, supporting the translational relevance of insights derived from *Drosophila* studies to mammalian reproductive biology.

On the other hand, CySCs in *Drosophila* testis exhibit slightly different metabolic features. The fluorescent reporter Mitotimer shows a low oxidative state in the mitochondrial matrix of CySCs. Knocking out *Pdk* (Pyruvate dehydrogenase kinase) in CySCs inhibits the phosphorylation of the pyruvate dehydrogenase complex, forcing pyruvate to enter the mitochondria for OXPHOS. This disrupts a glycolytic mode required for CySCs to maintain their stem cell state, leading to CySC loss and depletion [23]. These findings suggest that CySCs exhibit a glycolytic-biased metabolic mode. In contrast, the Mitotimer sensor shows that mitochondrial oxidation level in cyst cells is high. Furthermore, transcriptome sequencing data shows that, compared to CySCs, the expression of genes related to the mitochondrial TCA cycle and electron transport chain (ETC) in cyst cells is significantly upregulated. Specifically, the expression profile of key genes regulating pyruvate metabolism is reversed. The expression of *Pdk* is downregulated, while its antagonist, *Pdp* (Pyruvate decarboxylase phosphatase), is correspondingly upregulated in cyst cells. This evidence indicates that cyst cells overall exhibit a metabolic pattern dominated by mitochondrial OXPHOS. However, although cyst cells are predominantly oxidative, glycolysis is still retained and used for lactate production. LDH (Lactate dehydrogenase) is a key enzyme that mediates the conversion of pyruvate to lactate and is commonly used as a metabolic marker of enhanced lactate-producing glycolysis. Notably, *LDH* is significantly upregulated in cyst cells [23]. Pdk inhibits the activity of Pdh by phosphorylation, thereby ensuring that glycolysis-derived pyruvate is preferentially converted into lactate rather than entering the mitochondria. Importantly, lactate is shuttled to enclosed germ cells via the monocarboxylate transporter Milkman, supporting germ cell survival [10]. This metabolic support depends on the formation of septate junctions between cyst cells, which create a specialized microenvironment permissive for somatic-to-germline metabolite shuttling. Moreover, studies in the *Drosophila* ovary demonstrate that somatic follicle cells import amino acids via transporters such as the monocarboxylate transporter Cochonnet and that amino acids entering follicle cells are subsequently transferred to germ lineage cells through Inx2 (Innexin 2)/Inx4-dependent gap junctions at the soma–germline interface, thereby promoting germ cell growth [24]. Disruption of either gap junction formation or amino acid import in somatic cells induces P-body (Processing body) accumulation and translational repression in germ cells, functionally linking soma-derived amino acids to germline growth control. Although the precise amino acid species and individual germ cell types, which directly receive these metabolites, remain unresolved, the metabolic support is clearly exerted at the level of germline as a whole. Together with the cyst-derived lactate shuttle described in *Drosophila* testis, these findings support a conserved principle whereby somatic cells preferentially execute metabolically demanding import or synthesis steps and subsequently distribute key metabolites to germ lineage cells. Importantly, in addition to sustaining their own stem cell identity, this observation suggests that somatic cyst lineage cells contribute to the metabolic support of developing germ cells.

Up to now, direct metabolic interactions between CySCs and GSCs in *Drosophila* testis niche have not yet been well characterized, and whether these two stem cell populations engage in direct metabolic coupling remains an open question. In the mouse intestinal stem cell (ISC) niche, Paneth cells function as specialized niche support cells that preferentially engage in glycolysis and produce lactate, whereas ISCs rely on mitochondrial OXPHOS and utilize lactate as an oxidative fuel [25]. Although Paneth–ISC interactions cannot be directly equated with CySC–GSC relationships in *Drosophila* testis, this paradigm may provide a conceptual framework in which support cells regulate stem cell function through the supply of metabolic substrates.

### 2.2. Mitochondrial Structure and Function in GSCs

Mitochondria serve as a metabolic hub integrating energy production, biosynthesis, and signaling in stem cells. In *Drosophila* testis stem cells, mitochondrial morphology, distribution, and function are precisely controlled by the balance of the mitochondrial fusion proteins Marf (*Drosophila* ortholog of Mitofusin 2) and Optic atrophy 1 (Opa1) and the fission protein Dynamin-related protein 1 (Drp1). Beyond ATP generation, mitochondria play crucial roles in reactive oxygen species (ROS)-mediated signaling for stem cell homeostasis, production of epigenetic-modulating metabolites such as TCA intermediates and lipids, as well as integration of cell-intrinsic signals such as TOR, which regulates stem cell fate and lipid catabolism in cyst lineage cells [12,26].

GSCs typically contain a fewer number of mitochondria with more fragmented morphology compared to differentiating germ cells, which generally accumulate elongated, interconnected mitochondrial networks [26]. Genetic studies have demonstrated that disrupting mitochondrial dynamics (fusion/fission) profoundly affects stem cell behavior [27]. For example, in the male testis, disruption of Drp1-mediated mitochondrial fission leads to differentiation-driven depletion of GSCs. Specifically, loss of Drp1 function results in excessive mitochondrial fusion and a concomitant increase in ROS levels. Elevated ROS subsequently upregulates the EGF ligand Spitz and activates EGFR signaling in adjacent somatic cyst cells. Aberrant activation of EGFR signaling ultimately drives premature differentiation of GSCs, leading to stem cell loss [26]. In support of this, mutant clonal analysis of *Drp1* using FLP/FRT recombination showed strong phenotypes, including the total loss of GSCs and spermatogonia in larval stages. Furthermore, ectopic expression of dominant-negative *Drp1* using *nanos*-Gal4 driver, which is expressed in germ lineage cells, significantly impaired mitochondrial fission and reduced GSC numbers in 1-day-old adults, while RNAi-mediated *Drp1* knockdown yielded milder effects due to its incomplete depletion [26]. In contrast, studies in the *Drosophila* ovary have demonstrated that altered mitochondrial dynamics are closely linked to age-dependent GSC decline. In the natural aging process of the *Drosophila* ovary, mitochondrial dynamics in GSCs shift toward increased fission, accompanied by reduced BMP signaling, decreased mitochondrial membrane potential, and age-dependent stem cell loss. Consistently, forced mitochondrial fragmentation experimentally recapitulates the features of aged GSCs, whereas genetic suppression of *Drp1*-mediated fission attenuates GSC loss during aging [27]. Notably, male GSCs also undergo age-associated decline under physiological conditions, characterized by reduced cell cycle activity, a decrease in hub cell number, and a progressive loss of GSCs [28]. However, whether mitochondrial dynamics contribute to aging-associated changes in male GSCs remains largely unexplored. In summary, these findings suggest that mitochondrial structure and function are closely associated with the metabolic demands of GSCs and contribute to the regulation of their fate and homeostasis.

Mitochondrial ROS production plays a dual role in stem cell regulation [29]. Moderate levels of ROS are required for the maintenance of stem cells in the *Drosophila* testis. However, low levels of ROS induced by knockdown of *Keap1* or ectopic expression of *CncC* (*Drosophila* ortholog of *Nrf2*) cause an overgrowth of early-stage germ cells [30]. By contrast, excessive ROS levels result in oxidative stress to macromolecules such as proteins and DNA, impairing stem cell behavior [31]. Furthermore, oxidative stress in germ cells leads to the activation of the EGFR signaling pathway in cyst lineage cells, which promotes premature differentiation of GSCs [30]. While mitochondrial dynamics and ROS signaling have been extensively characterized, the mechanisms linking specific mitochondrial morphologies to metabolic outputs remain incompletely understood. Indeed, most studies infer metabolic changes from mitochondrial structure but lack direct measurements of respiratory capacity or metabolite production in cells with altered mitochondrial dynamics [32]. The molecular mechanisms underlying asymmetric mitochondrial inheritance during stem cell division are also poorly defined.

### 2.3. Nutrient Sensing and Dietary Regulation of Testis Stem Cell Behavior

*Drosophila* testis stem cells are exquisitely sensitive to organismal nutritional status, enabling reproductive output to be matched with available resources [33]. This coupling between metabolism and reproduction is mediated by the evolutionarily conserved nutrient-sensing pathways, including insulin/insulin-like growth factor signaling (IIS), TOR, and AMP-activated protein kinase (AMPK) [34]. Under nutrient-rich conditions, circulating insulin-like peptides bind to insulin receptor (InR) on stem cells, activating downstream PI3K–Akt cascade, thereby regulating organismal growth and metabolic homeostasis [35]. Hence, the insulin/TOR pathway has been shown to play a central role in translating nutritional information into stem cell behavior in *Drosophila* testis [36].

Lipid metabolism and lipid-derived signals also influence testis stem cell behavior. Studies have shown that lipid metabolism critically regulates GSC maintenance in the *Drosophila* testis, as mitochondrial dysfunction impairs fatty acid oxidation, leading to lipid droplet accumulation, aberrant TOR–SREBP (Sterol Regulatory Element-Binding Protein) activation, and stem cell loss [26]. Lipid metabolism also plays a critical role in regulating the behavior of CySCs. Studies also reveal that knockdown of autophagy-related genes such as *Atg1* (Autophagy-related 1) or *Atg8a*, or inhibition of EGFR signaling in CySCs and early cyst cells, disrupts lipid metabolic homeostasis. The impaired lipid metabolism is manifested by aberrant lipid droplet accumulation and consequently impairs the function and proper differentiation of CySCs and their progeny [37].

Notably, studies in multiple mammalian systems have shown that mTORC1 (mammalian Target of Rapamycin Complex 1) is dynamically regulated by diverse environmental inputs, including amino acid availability. Among these, leucine and arginine play prominent roles through Rag GTPases and their associated regulatory complexes such as GATOR complex to coordinate cellular growth and metabolic states with environmental conditions [38]. Moreover, studies in *Drosophila* S2 cultured cell models have shown that treatment with amino acids that directly activate the TOR pathway induces downstream responses, including S6K-dependent Myc accumulation [39]. Together, these findings support the notion that TOR functions as a conserved nutrient sensor that links amino acid availability to the regulation of cellular growth and metabolic programs. Although direct evidence for amino acid-mediated regulation of TOR signaling in *Drosophila* testis is currently lacking, whether and how amino acids modulate TOR signaling in this tissue, and how such regulation impacts stem cell metabolism and fate decisions, remain key unanswered questions that warrant further investigation.

### 2.4. Specialized Metabolic Pathways

The stem cell niche functions not only as a structural scaffold and signaling hub but also as an active metabolic microenvironment in which cells interact metabolically through the secretion of regulatory factors. These ligands activate distinct signaling pathways in neighboring GSCs and CySCs that couple stem cell identity maintenance with metabolic state. For instance, Unpaired (Upd; IL-6-like cytokine) secreted by hub cells activates JAK-STAT signaling in both GSCs and CySCs. The survival and maintenance of these two stem cell populations depend on this signaling, thereby ensuring the structural integrity of the stem cell niche [5]. Also, the BMP ligands, Decapentaplegic (Dpp) and Glass bottom boat (Gbb) secreted by hub cells and CySCs activate BMP signaling in adjacent GSCs, thereby facilitating GSC maintenance.

Recent work also suggests that hub cells might buffer stem cells from metabolic perturbations, maintaining a relatively stable metabolic niche even when systemic conditions fluctuate [40]. However, to date, most evidence for metabolic coupling in the *Drosophila* testis has been indirectly derived from differential metabolic profiles of distinct cell types or phenotypes of metabolic mutants; direct demonstrations of metabolite transfer between defined cell types still remain scarce [41]. The spatial organization of metabolism within the niche and how metabolite gradients are established remain poorly understood. It also remains challenging to distinguish relative contributions of cell-autonomous versus non-cell-autonomous metabolic regulation within this system.

## 3. Signaling Pathways Regulating Stem Cell Metabolism

**Figure 2:**
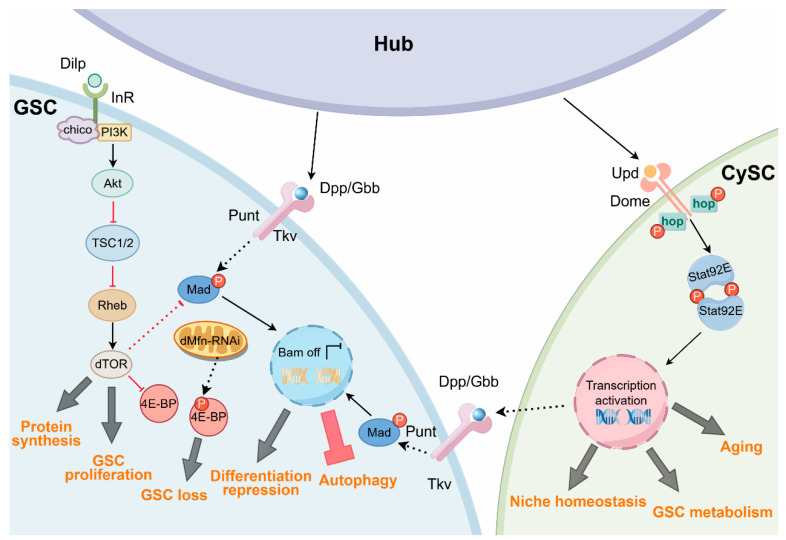
Schematic of the metabolic signaling pathways in GSCs and CySCs. The JAK/STAT pathway in CySCs not only regulates their own aging process but also maintains niche homeostasis and establishes a microenvironment permissive for proper GSC division through non-cell-autonomous mechanisms. The insulin/TOR pathway in GSCs promotes GSC growth via protein synthesis. However, mitochondrial dysfunction induced by *dMfn* inhibition causes an ectopic activation of the TOR signaling pathway, leading to the loss of GSCs. Moreover, acting as a signaling integration node between the insulin/TOR and BMP pathways, dTOR can indirectly modulate GSC self-renewal capacity. Sustained BMP signaling in GSCs suppresses Bam expression to prevent premature differentiation and is associated with reduced autophagy levels in GSCs.

The BMP, JAK-STAT, and Insulin/TOR signaling pathways converge within the *Drosophila* testis stem cell niche to coordinately regulate the metabolic state of stem cells (Figure 2).

### 3.1. BMP/TGF-β Signaling and Metabolic Control

The BMP pathway is the predominant niche signal required for the maintenance of GSCs in *Drosophila* testis and has recently been recognized to exert significant metabolic effects [42]. Hub cells secrete Dpp and Gbb, which bind to receptors on adjacent GSCs, activating Mothers against dpp (Mad), the *Drosophila* Smad1/5 homolog [43]. Phosphorylated Mad in GSCs translocates to the nucleus, where it regulates the transcription of various target genes. For instance, the suppression of Bag of marbles (Bam), a key differentiating factor, is essential for maintaining GSC stemness by preventing differentiation [4].

In the testis stem cell niche, BMP signaling not only governs self-renewal of GSCs through the repression of Bam but is increasingly recognized to intersect with metabolic regulation. For example, the BMP–Bam axis suppresses autophagy in GSCs (Figure 2) while promoting autophagy in differentiating germ cells, thereby ensuring that changes in metabolic state are coordinated with cell fate transitions during differentiation [44]. Furthermore, BMP signaling appears to interact with the nutrient-sensing TOR signaling pathway, which is required in GSCs and early germ cells for timely differentiation. Genetic studies demonstrate that the loss of mTORC1 via *Raptor* knockdown leads to an abnormal upregulation of BMP signaling, as evidenced by a significant increase in nuclear phosphorylated Mad (pMad) levels (Figure 2) [45]. This indicates that mTORC1 acts as a negative regulator of BMP signaling in male germline cells. Consequently, the absence of mTORC1 hinders or delays GSC differentiation, resulting in an accumulation of germ cells with GSC-like features. These observations may suggest that nutritional signals can modulate the sensitivity of GSCs to BMP-mediated self-renewal cues. Additionally, there is evidence that insulin signaling and the BMP pathway converge on common targets in mammalian systems. For example, in mouse brown preadipocytes, BMP7 modulates IRS (Insulin Receptor Substrate)/PI3K/Akt activity for robust brown adipogenesis, and insulin pathway activity is also necessary for a full BMP7 response [46]. Although a direct molecular convergence between insulin/TOR and BMP signaling has not been demonstrated in *Drosophila* testis, these pathways functionally intersect at the level of GSC behavior. In support of this, loss of mTORC1 activity has been shown to block or delay GSC differentiation, and BMP signaling has greatly been upregulated in GSCs and early germ cells, resulting in an accumulation of early-stage germ cells [45]. This crosstalk suggests that BMP may influence metabolic pathways, including mTOR, to integrate external niche signals with intracellular metabolic state and thereby regulate stem cell fate. In addition, in other biological systems such as mouse models, BMP family members have been shown to modulate mitochondrial biogenesis and OXPHOS by controlling the expression of metabolic regulators such as OXPHOS components, highlighting a potential conserved link between the BMP pathway and mitochondrial function [47]. This bidirectional relationship suggests the existence of feedback mechanisms that couple metabolic status to niche signaling [48]. While transcriptional effects of BMP signaling on metabolic genes are increasingly recognized, direct measurements of metabolic flux changes in response to BMP signaling manipulation are lacking [49]. Most studies infer metabolic changes from gene expression data, but transcript levels do not always correlate with enzyme activity or pathway flux. The mechanisms through which BMP signaling regulates specific metabolic pathways at the molecular level still remain largely unknown.

### 3.2. JAK-STAT Signaling and Metabolic Adaptation in Testis Stem Cells and Adult Muscle

The JAK-STAT pathway is critical for maintaining CySCs and also plays important roles in GSC self-renewal, particularly under stress conditions [50]. In the testis stem cell niche, the JAK-STAT ligand Upd is produced by hub cells and is required for self-renewal of both GSCs and CySCs [51]. For instance, studies demonstrate that the aging process interferes with the specialized division mechanism in GSCs, preventing them from properly detaching from their progeny cells. Through comprehensive live-cell imaging and genetic studies, it has been shown that this detachment issue arises from the abnormal persistence of F-actin within the cytoplasmic bridge linking the stem cell to its daughters. The regulation of F-actin occurs via the JAK-STAT pathway, where elevating or reducing its activity can accordingly mitigate or worsen age-related separation problems. Notably, even a slight decrease in STAT activity can induce premature aging in GSCs, resulting in failed separation [52]. In addition, the JAK/STAT pathway regulates cell fate conversion through Esg (Escargot)-mediated inhibition of hub-to-CySC conversion [53] while also controlling cell competition by inhibiting MAPK [54], thereby achieving the maintenance of stem cell niche homeostasis and preventing excessive differentiation and depletion of stem cells. What is more, the JAK/STAT pathway has emerged as a key regulator of metabolic adaptation in *Drosophila* intestinal stem cells, enabling them to respond to various stressors, including nutrient deprivation, oxidative stress, and aging [55].

The JAK/STAT signaling pathway also plays a key role in the transcriptional regulation of metabolic genes in *Drosophila*, primarily by responding to dietary and infection stresses to modulate insulin signaling and nutrient allocation, thereby maintaining metabolic homeostasis. Under a high-fat diet, this pathway becomes activated by macrophage-derived Upd3, leading to a sustained inhibition of insulin signaling, hyperglycemia, an increase in fat storage, and a shortened lifespan [56]. In adult muscles, basal JAK/STAT signaling inhibits Akt and regulates FoxO target genes to maintain energy stores (such as triglycerides and glycogen) and insulin balance, avoiding elevated metabolic rates and muscle dysfunction [57]. During parasitic infection, hemocyte-secreted Upd2/3 activates muscle JAK/STAT signaling, which mutually feedbacks with the insulin pathway to regulate carbohydrate metabolism (such as muscle glycogen consumption and elevated blood sugar), feeding behavior, and a systemic Dilp (*Drosophila* insulin-like peptide) expression, supporting nutrient redirection under immune responses. Furthermore, infection results in JAK-STAT activation to transcriptionally induce the insulin antagonist ImpL (Imaginal morphogenesis protein-Late), promoting adaptive insulin resistance and transferring nutrients from muscles to immune cells while delaying development to ensure homeostasis [58,59]. Overall, the JAK-STAT pathway integrates immunity and metabolism under non-inflammatory and stress conditions, conservatively regulating genes to cope with energy demands.

### 3.3. Insulin/TOR Signaling Integration

The insulin/TOR signaling pathway has been well established as a highly conserved metabolic pathway across species. It responds to extracellular Dilps, intracellular amino acids, and energy status to coordinate the growth, proliferation, and metabolism of stem cells. InR activation triggers a signaling cascade involving InR substrate (Chico), PI3K, and Akt kinases [60]. Akt phosphorylates downstream targets, including mTORC1, which in turn regulates metabolic processes and FoxO activity [61]. Activated mTORC1 then phosphorylates S6 kinase (S6K) and 4E-binding protein (4E-BP) to promote protein translation and ribosome biogenesis [62].

The insulin/TOR signaling network serves as a primary integrator of nutritional information and metabolic status in testis stem cells [36]. In the *Drosophila* testis niche, mTORC1 regulates metabolism-linked cellular behaviors in both GSCs and CySCs, including cell growth, proliferative capacity, and the progression of cell differentiation [45]. For example, inhibition of mTOR kinase induces a delay or arrest in germ cell differentiation [45]. Another example is the fact that in early germ cells, RNAi-mediated depletion of *dMfn* results in mitochondrial dysfunction, ectopic activation of the TOR signaling pathway, aberrant phosphorylation of 4E-BP, and subsequent loss of GSCs (Figure 2). Additionally, CySC differentiation is actively promoted by PI3K/TOR signaling, whereas CySCs lacking PI3K/TOR activity fail to undergo proper differentiation [36]. These lines of evidence indicate that mTOR signaling plays a critical role in the maintenance of both GSC and CySC homeostasis. On the other hand, in mammals, mTORC1 functions as a central metabolic hub integrating insulin signaling, amino acid availability, and energy status to drive anabolic metabolism and suppress autophagy [38]. However, in *Drosophila*, although mTORC1 has been shown to regulate stem cell behavior, its direct roles in controlling metabolic pathways in the male germline remain largely unexplored. Furthermore, despite extensive understanding of core components and general roles of the insulin/TOR pathway, its precise mechanisms in regulating specific metabolic processes within testis stem cells, as opposed to other cell types, remain poorly defined. Moreover, temporal dynamics of insulin/TOR activity under acute nutritional fluctuations are largely unexplored, as most existing studies have focused on chronic dietary restriction or long-term genetic perturbations.

## 4. Emerging Technologies to Understand Metabolic Regulation in Testis Stem Cells

### 4.1. Single-Cell Metabolomics and Spatial Metabolomics

Traditional metabolomics measures all metabolites in a tissue sample at once, indicating that it is feasible to lose information about which specific cells have which metabolites [41]. Single-cell metabolomics can solve this problem by measuring metabolites in individual cells with current technology [63]. Mass spectrometry imaging can detect hundreds of metabolites in intact tissues with a very high spatial resolution, allowing researchers to create detailed maps showing exactly where each metabolite is located [64]. When applied to the testis, spatial metabolomics will be able to reveal metabolic differences between the stem cell niche center and the surrounding stem cell populations, helping us understand how stem cells maintain a special metabolic environment. Single-cell metabolomics also reveals metabolic differences between individual stem cells that would be hidden in traditional bulk measurements [65]. Spatial metabolomics would thus show the metabolic microenvironment surrounding stem cells. Together, these technologies are expected to help us understand the complete picture of stem cell metabolism. While single-cell metabolomics is promising, we still lack complete information about all metabolites in a single cell because individual cells contain very small amounts of metabolites that could break down quickly [66]. Many metabolites cannot be detected yet due to technical limitations, and it takes time to analyze the data. Development of faster and more sensitive methods to measure metabolites in intact organs like the testis is needed.

### 4.2. Artificial Intelligence and Machine Learning in Stem Cell Metabolism Research

Artificial intelligence (AI) and machine learning (ML) are transforming biological research, including stem cell metabolism, by enabling analysis of complex, high-dimensional datasets [67]. Deep learning approaches are being applied to predict cellular metabolic states from morphological or transcriptomic features. For example, a deep learning algorithm (MoDL) trained on super-resolution images of mitochondria can accurately predict mitochondrial functions by analyzing morphological features, demonstrating that ML can link subcellular structure to metabolic function at a single-organelle resolution [68]. Moreover, in broader single-cell studies, transcriptomic data remain the most commonly used data type. While static transcriptomic data reflect the abundance of metabolic enzyme mRNAs, dynamic metabolic fluxes or actual biochemical activities cannot be directly captured due to post-transcriptional regulation and network-level constraints [69]. GEFMAP (Gene Expression-based Flux Mapping and Metabolic pathway Prediction) leverages metabolic network topology to partially address these limitations, enabling the inference of relative metabolic flux patterns from single-cell transcriptomic profiles [70]. These advances indicate that deep learning is a powerful tool for connecting a cell’s molecular and structural information with its functional metabolic state. Furthermore, convolutional neural networks (CNNs) can analyze microscopy images of cells to predict their metabolic profiles without requiring destructive metabolic measurements, enabling high-throughput metabolic classification and screening [71]. For example, AI models trained on images of cells with known metabolic states can predict whether stem cells are glycolytic or oxidative, identify cells with mitochondrial dysfunction, or detect metabolic stress—all from simple brightfield or fluorescence images [72]. Applied to the *Drosophila* testis, such approaches could enable non-invasive, longitudinal monitoring of stem cell metabolic states in vivo.

Despite the excitement about AI applications, there are still significant challenges. ML models are often “black boxes,” lacking interpretability, meaning that they make accurate predictions but provide limited mechanistic insights [73]. Models trained on specific datasets may not generalize to other contexts, especially across species or experimental conditions in biology [74]. The quality of AI predictions strictly depends on the quality and quantity of training data, which may be limited for specialized systems such as the *Drosophila* testis. Although *Drosophila* testis-specific datasets are currently limited, transfer learning may provide a potential solution to this constraint. Studies have shown that it can overcome this challenge by leveraging knowledge from related biological systems. For instance, a deep learning model pre-trained on large-scale transcriptomic data comprising 30 million single cells can be fine-tuned with as few as 884 tissue-specific samples to achieve accurate predictions [75]. A robust transfer learning method named Trans-PtLR demonstrates that it maintains high performance even when handling heterogeneous data from different sources [76]. This approach enables AI-driven metabolic predictions in testis stem cells by transferring knowledge from mammalian stem cells or other *Drosophila* tissues, demonstrating both current feasibility and significant future potential.

## 5. Conclusions and Perspectives

Metabolism has emerged from a supporting role to a center stage in stem cell biology in recent years. The *Drosophila* testis, with its accessible, well-characterized architecture, has provided fundamental insights into how metabolic regulation controls stem cell fate decisions, niche interactions, and even responses to aging and stress. Stem cells are not simply cells that happen to have particular metabolic profiles; rather, metabolism actively instructs stem cell identity and behavior through multiple mechanisms, including energy provision, biosynthetic precursor supply, signaling metabolite production, and epigenetic regulation [77]. Several key principles have emerged from studies of testis stem cell metabolism. First, GSCs exhibit distinctive metabolic states that involve tightly regulated mitochondrial dynamics and redox homeostasis [30,78]. Second, major developmental signaling pathways, including BMP, JAK-STAT and insulin/TOR, extensively regulate metabolism while being reciprocally influenced by metabolic state. Third, stem cells exist within metabolically active niches where metabolic coupling between different cell types coordinates their behavior. Fourth, metabolic dysfunction serves as a cause and consequence of stem cell aging, while metabolic interventions can preserve stem cell function.

Several key research directions should be followed. First, comprehensive spatial metabolomics mapping of the testis niche should be conducted to track how metabolite distributions change across different dietary regimens, environmental stresses, and aging conditions, thereby elucidating how these metabolic dynamics influence stem cell behavior. Second, it is necessary to develop a deeper understanding of the metabolic coupling mechanisms between niche cells and adjacent stem cells, revealing how they communicate each other through metabolite exchange. Third, AI-guided discovery of metabolic interventions should be accelerated to rapidly identify novel compounds and therapeutic strategies that protect stem cells. Research on metabolic regulation of *Drosophila* testis stem cells exemplifies the power of a simple, genetically tractable model system in generating fundamental discoveries with broad implications. With continuous advances in new technologies, including single-cell sequencing, real-time imaging, and AI, together with increasingly sophisticated interdisciplinary research approaches, rapid progress in understanding how metabolism controls stem cell behavior can be expected. Knowledge gained from these model systems will ultimately guide rational design of metabolic intervention strategies to maintain stem cell function throughout the human lifespan. This research may have profound implications for reproductive health, aging research, and regenerative medicine, potentially opening new avenues for treating age-related fertility decline and developing novel regenerative therapies.

## Data Availability

No datasets were generated or analyzed during the current study. Data sharing is not applicable.
